# Supplementary material for: Saccharomyces boulardii (CNCM I-745) improves intestinal damage in sepsis by remodeling intestinal flora structure
Source: Front Cell Infect Microbiol. 2025 Jul 31;15:1602792. doi: 10.3389/fcimb.2025.1602792 (PMC12350329; doi:10.3389/fcimb.2025.1602792)
Supplement: Supplementary file 1 [file DataSheet1.zip › summary/summary/8_Advanced_analyse/2_PICRUSt2_result/COG/Group.DW4_vs_DW0/PICRUSt2_COG_Group.DW4_vs_DW0_diff.pdf]

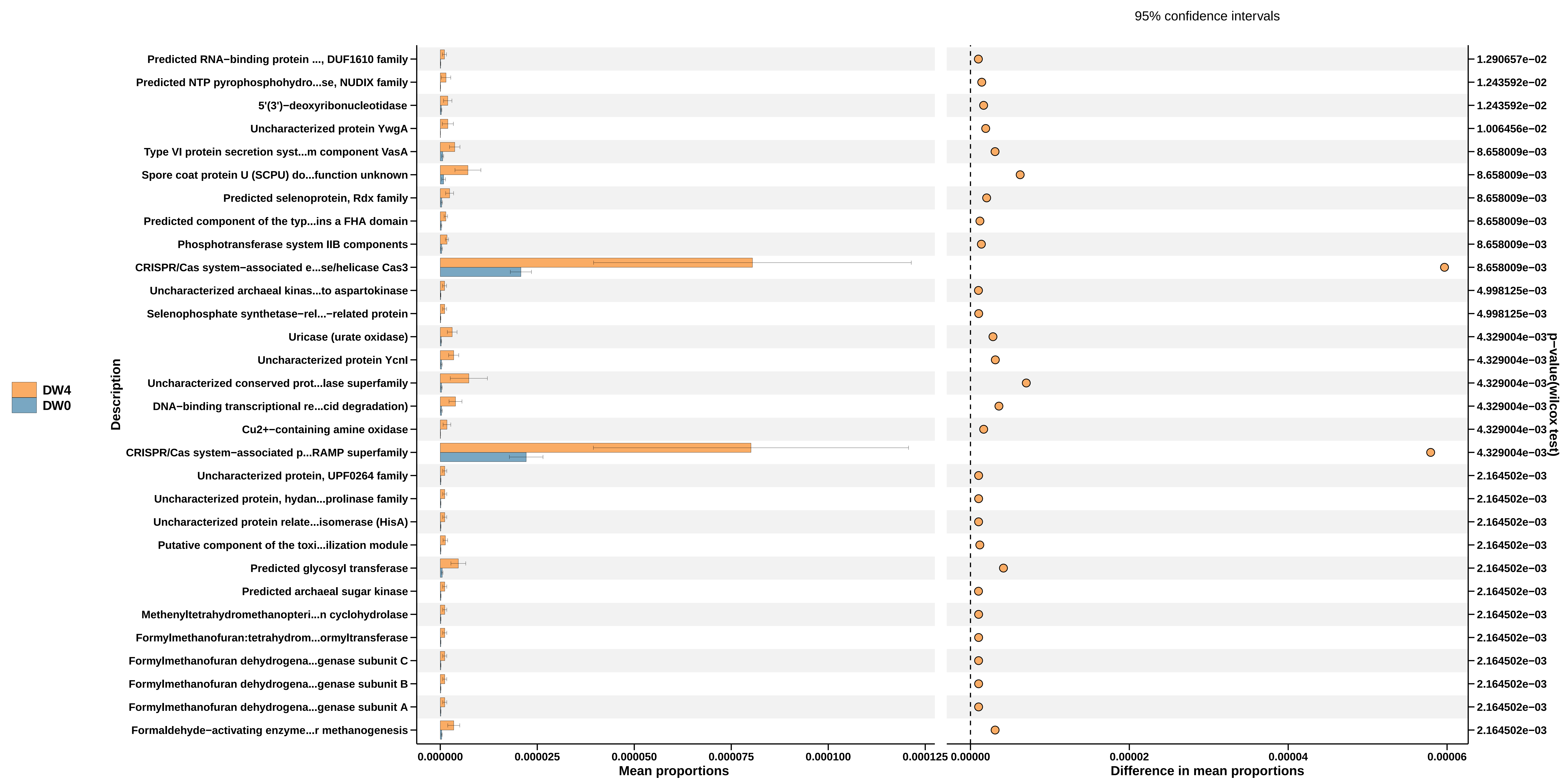
95% confidence intervals
DW4
DW0
Description
Predicted RNA−binding protein ..., DUF1610 family
Predicted NTP pyrophosphohydro...se, NUDIX family
5'(3')−deoxyribonucleotidase
Uncharacterized protein YwgA
Type VI protein secretion syst...m component VasA
Spore coat protein U (SCPU) do...function unknown
Predicted selenoprotein, Rdx family
Predicted component of the typ...ins a FHA domain
Phosphotransferase system IIB components
CRISPR/Cas system−associated e...se/helicase Cas3
Uncharacterized archaeal kinas...to aspartokinase
Selenophosphate synthetase−rel...−related protein
Uricase (urate oxidase)
Uncharacterized protein YcnI
Uncharacterized conserved prot...lase superfamily
DNA−binding transcriptional re...cid degradation)
Cu2+−containing amine oxidase
CRISPR/Cas system−associated p...RAMP superfamily
Uncharacterized protein, UPF0264 family
Uncharacterized protein, hydan...prolinase family
Uncharacterized protein relate...isomerase (HisA)
Putative component of the toxi...ilization module
Predicted glycosyl transferase
Predicted archaeal sugar kinase
Methenyltetrahydromethanopteri...n cyclohydrolase
Formylmethanofuran:tetrahydrom...ormyltransferase
Formylmethanofuran dehydrogena...genase subunit C
Formylmethanofuran dehydrogena...genase subunit B
Formylmethanofuran dehydrogena...genase subunit A
Formaldehyde−activating enzyme...r methanogenesis
0.000000
0.000025
0.000050
0.000075
0.000100
0.000125
Mean proportions
0.00000
0.00002
0.00004
0.00006
Difference in mean proportions
p−value(wilcox test)
1.290657e−02
1.243592e−02
1.243592e−02
1.006456e−02
8.658009e−03
8.658009e−03
8.658009e−03
8.658009e−03
8.658009e−03
8.658009e−03
4.998125e−03
4.998125e−03
4.329004e−03
4.329004e−03
4.329004e−03
4.329004e−03
4.329004e−03
4.329004e−03
2.164502e−03
2.164502e−03
2.164502e−03
2.164502e−03
2.164502e−03
2.164502e−03
2.164502e−03
2.164502e−03
2.164502e−03
2.164502e−03
2.164502e−03
2.164502e−03
